# Seeing emotions: an eye-tracking study of emotion recognition in deaf individuals amid facial occlusions

**DOI:** 10.3389/fpsyg.2025.1496259

**Published:** 2025-05-16

**Authors:** Yu Chen, Shengqin Cao, Zhiquan Zhou, Kaiwen Cheng

**Affiliations:** ^1^School of Foreign Languages, Southeast University, Nanjing, China; ^2^Technical College for the Deaf, Tianjin University of Technology, Tianjin, China; ^3^National Research Center for Language and Well-Being, Shanghai Jiao Tong University, Shanghai, China; ^4^College of Language Intelligence, Sichuan International Studies University, Chongqing, China

**Keywords:** deaf individuals, emotion recognition, occlusion, integrative hypothesis of visual function, eye-tracking

## Abstract

Extensive research has demonstrated that facial occlusion significantly affects individuals’ emotion recognition abilities. However, whether facial occlusion exacerbate the difficulty in emotion recognition for deaf individuals remains elusive. This study employed eye-tracking technology to investigate the mechanisms underlying emotion perception in deaf individuals under different facial occlusion conditions. We compared the percentage of eye and mouth gaze fixation in deaf and hearing participants as they judged different emotions (positive, neutral, negative) under three occlusion conditions (no occlusion, sunglasses, mask). The behavioral and eye-tracking results reveal that, first, facial occlusion by sunglasses and mask significantly impairs emotion perception and social communication for deaf individuals. Second, the eye area is more crucial for recognizing negative emotions, while the mouth area is critical for recognizing positive emotions. Third, deaf individuals exhibit a “happiness superiority effect,” responding more favorably to positive emotions and showing an avoidance bias toward negative emotions. Besides, visual attention allocation strategies of deaf individuals tend to be relatively fixed and less adaptable to task demands. Overall, these findings support the integrative hypothesis of visual function in deaf individuals and provide insights for enhancing facial emotion recognition and optimizing social interaction strategies for the deaf community.

## 1 Introduction

Facial expressions, the primary means by which emotions are conveyed, serve as essential external manifestations of emotional states. These expressions provide critical clues about an individual’s internal state, significantly impacting social abilities and forming a crucial foundation for establishing complete and effective social interactions (Liu and Ge, 2014). Studies on the fixation patterns of emotional faces have shown that when viewing different emotional images, there may be significant differences in the fixation proportions on different facial regions. That is, different areas of interest may play dominant roles in conveying different emotions. For instance, numerous research indicates that eyes are the most important area for recognizing negative emotions, including sadness and fear, while the mouth is more closely associated with positive emotions, such as happiness (Beaudry et al., 2014; Bombari et al., 2013; Schurgin et al., 2014). However, research by Blais et al. (2012) demonstrated that the mouth region provided critical cues for eight static and dynamic facial expressions (including six basic emotions, pain, and neutral expressions), suggesting that while specific facial regions have important influences on specific emotions, emotion recognition relies on other facial regions as well. Additionally, Nummenmaa and Calvo (2015) found that happy faces were recognized more quickly and accurately than other expressions in emotion classification tasks. This finding aligns with the importance of positive emotions in social interactions, as happy expressions typically convey positive social signals, making them easier to recognize and respond to.

Currently, the use of facial occlusions, such as sunglasses and mask, has become increasingly prevalent due to health concerns (Rabadan et al., 2022), esthetic preferences (Harris, 1991), and occupational requirements. However, such occlusions can significantly impair the recognition of emotions, reducing the identifiability of facial expressions and the accuracy of emotion recognition (Carbon, 2020; Ruba and Pollak, 2020). Sunglasses, which obscure the eyes, the crucial feature for expressing and recognizing emotions, can diminish the overall communicative effectiveness of facial expressions, affecting the accuracy and speed of emotion recognition. Studies by Kramer and Ritchie (2016) and Graham and Ritchie (2019) support these findings, indicating that sunglasses impair observers’ ability to match faces and reduces trust in the observed individuals. Mask, another common type of facial occlusion, obscures the lower part of the face, including the mouth, an area crucial for conveying and recognizing emotions. Since the outbreak of the COVID-19 pandemic, the frequency of mask-wearing has increased significantly. Besides, some professionals, including healthcare workers and construction workers, often wear mask and protective gear to protect against viruses, dust, or chemicals. Recent studies confirm that masks have a negative impact on the ability to recognize emotions (Parada-Fernandez et al., 2022; Pazhoohi et al., 2021). Grahlow et al. (2022) discovered that the capability to identify emotions in masked faces was impaired across all six emotional conditions. Mask-wearing reduces the perceived intensity of emotions, thereby diminishing individuals’ ability and confidence in recognizing facial expressions (Kastendieck et al., 2022; Pazhoohi et al., 2021). Malak and Yildirim (2022) conducted an eye-tracking investigation which revealed that the presence of masks significantly diminishes the accuracy of recognizing fearful facial expressions, without exerting a similar impact on the perception of angry or neutral faces. The neural underpinnings of these findings were further examined by Prete et al. (2022) through electroencephalogram (EEG) analysis. This research posited that masked faces could act as anxiety-provoking stimuli, thereby impairing the efficiency and efficacy of emotional recognition processes. Such impairment was evidenced by increased N170 amplitudes—a marker indicative of early stages of face processing—as well as decreased P2 amplitudes, which are associated with subsequent higher-level cognitive processing stages. These insights are substantiated by complementary behavioral and functional magnetic resonance imaging (fMRI) studies from Abutalebi et al. (2024), suggesting that the use of masks imposes additional demands on the mechanisms responsible for facial emotion recognition, affecting both the speed of this process and the allocation of neural resources involved.

Due to the absence of auditory input, the deaf community faces distinct challenges, such as increased communication difficulties, missed information, and heightened social disconnection in masked conditions (Gutierrez-Sigut et al., 2022; Mansutti et al., 2023). Despite these challenges, little research has examined the impact of facial occlusions on emotion recognition in deaf individuals. Generally, three key hypotheses explain the effects of auditory impairment on visual function in the deaf: the deficiency hypothesis, the compensation hypothesis, and the integration hypothesis (Ambert-Dahan et al., 2017; Alencar et al., 2019; Dye and Bavelier, 2010). The “deficiency hypothesis” posits that that auditory deprivation may disrupt the interplay among senses, leading to deficits in emotion perception (Ambert-Dahan et al., 2017; Esposito et al., 2017; Ludlow et al., 2010). Lau et al. (2022) assessed 59 deaf signers’ ratings of arousal, valence, invariant characteristics, and trait-like features of facial images under masked and unmasked conditions. Their findings revealed that while deaf signers perceive facial expressions more intensely than hearing individuals, they are also more inhibited by face masks. Amadeo et al. (2022) emphasized that face masks particularly impair the recognition of low-intensity expressions of happiness in deaf individuals. Furthermore, even when core linguistic comprehension in sign language remains intact, the ability to attribute emotions and attitudes is compromised when the lower face is obscured (Giovanelli et al., 2023). The “compensation hypothesis” posits that deaf individuals develop enhanced visual functions to compensate for the loss of auditory information (Alencar et al., 2019). This compensation may manifest as faster reaction times or an increased perceptual range in peripheral vision (Buckley et al., 2010). Supporting this idea, studies showed that deaf individuals often outperform hearing controls in target face matching tasks, suggesting a general visual processing advantage (Megreya and Bindemann, 2017).

In contrast, Rodger et al. (2021) discovered that individuals with early-onset severe deafness and hearing individuals performed similarly in recognizing six basic facial expressions, and dynamic stimuli did not provide an advantage over static expressions for the deaf. Dye and Bavelier (2010) proposed the “integration hypothesis,” which encompasses both the deficits and compensatory phenomena in the visual abilities of deaf individuals. Specifically, the changes in visual function for deaf individuals are dual-faceted: auditory deprivation can impair the development of the alerting network but can also enhance basic orienting functions, such as movement and engagement (Teresa Daza and Phillips-Silver, 2013), and spatial environment perception (Bell et al., 2019). Further supporting the complexity of these changes, attention network tests and brain network analyses by Ma et al. (2023) found that weakened fronto-occipital connectivity in the brains of deaf individuals altered alerting functions, but they might also acquire supplementary compensatory cognitive resources as a result of cortical inefficiency. Despite the insights, there is currently no definitive conclusion on how auditory loss affects the facial expression recognition abilities of deaf individuals.

Taken together, significant progress has been made in studying facial expression recognition, particularly the emotional perception abilities of hearing individuals. However, most research has focused on emotion recognition under no occlusion condition, neglecting common real-life scenarios involving facial occlusion, such as wearing sunglasses and mask. The impact of facial occlusion on emotion recognition has not been sufficiently studied. Several studies found that different types of facial occlusions (sunglasses, mask) significantly influenced the accuracy of emotion recognition, with the lowest recognition rates for images with mask, followed by sunglasses (Kim et al., 2022; Noyes et al., 2021). This impact might be more pronounced for the deaf population, who rely heavily on visual information for emotion perception (Amadeo et al., 2022). In light of this, the present study utilizes eye-tracking technology, using hearing individuals as a control group to examine the effects of different emotion types (positive, neutral, negative) and facial occlusion conditions (sunglasses, mask, original face) on facial emotion recognition in deaf individuals. We formulate two specific hypotheses. First, it is expected that facial occlusions (such as masks and sunglasses) will impair emotion recognition to a greater extent in deaf individuals compared to hearing individuals, given that deaf individuals rely more heavily on visual facial cues for emotional interpretation. Second, it is predicted that these occlusion effects would be emotion-specific, with sunglasses most severely disrupting recognition of negative emotions and masks most impairing recognition of positive emotions.

## 2 Methods

### 2.1 Participants

The experiment employed a 2 group (deaf people, hearing people) × 3 emotion (positive, neutral, negative) × 3 occlusion (sunglasses, mask, original face) multifactorial design. The group was a between-subjects variable, while emotion and occlusion were within-subjects variables. 63 participants were recruited for the experiment, including 30 deaf individuals (17 males and 11 females, with two males excluded—one due to heterogeneity concerns and another due to incomplete data) and 33 hearing individuals (13 males, 20 females). Based on a total sample size 61, a statistical power of 0.95, and an *α* level of 0.05, the G*Power 3.1 software (Faul et al., 2009) computed the effect size for the current study as 0.15.

All participants were undergraduate or master’s students from Tianjin University of Technology. They reported normal or corrected-to-normal vision, no color blindness or weakness, and no history of mental health issues or cognitive disorders. Before the experiment, participants gave written informed consent and filled out a basic information survey. As shown in Table 1, first, a total of 28 deaf participants were included, with 22 using hearing aids, one receiving a cochlear implant, and five with no experience using hearing devices. Second, started at 1.5 years on average, all deaf participants experienced severe to profound hearing loss with the mean hearing loss levels of 101.9 dB (SD = 32.6 dB, range: 60–207 dB) and 97.4 dB (SD = 15.8 dB, range: 65–120 dB) for the right ear and the left ear, respectively. Third, all deaf participants were native speakers of Chinese Sign language, while some of them frequently used Spoken Chinese in their daily lives. Since the study aimed to explore the overall impact of hearing loss on facial emotion recognition under occluded conditions, we did not strictly control language modality of the deaf participants.^1^ Meanwhile, none of the hearing participants in this study were sign language users. Upon completing the experiment, participants received compensation for their involvement. The study was approved by the Ethical Committee of Tianjin University of Technology.

**Table 1:** Questionnaire for relevant information of deaf subjects.

ID	Stimulation	Age of hearing loss (year)	Age at device using (year)	Left ear hearing loss(dB)	Right ear hearing loss(dB)	Frequency of spoken and sign language (%)	Dominant eye	Visual acuity (naked or corrected)
1	HA	0	3	100	120	30, 70%	L	4.2, 4.0
2	HA	4	4	110	70	95, 5%	R	4.3, 4.4
3	HA	2	12	120	89	70, 30%	L	5.1, 5.0
4	HA	1	3	89	89	0, 100%	L	4.6, 4.3
5	HA	0	8	95	95	0, 100%	L	4.5, 4.4
6	HA	3	19	110	110	20, 80%	L	4.3, 4.1
7	HA	5	7	65	70	80, 20%	L	5.0, 5.0
8	HA	1	6	65	60	30, 70%	R	5.0, 5.0
9	HA	1	23	90	93	20, 80%	L	4.8, 4.7
10	HA	2	2	110	123	60, 40%	R	4.9, 4.9
11	HA	0	2	120	120	60, 40%	R	5.0, 5.0
12	HA	1	5	100	120	30, 70%	R	5.0, 5.1
13	None	5	N	100	100	0, 100%	L	4.3, 4.0
14	HA	3	4	120	100	50, 50%	L	4.6, 4.5
15	HA	0	8	85	90	30, 70%	L	4.0, 4.0
16	CI	1	11	120	120	20, 80%	L	4.5, 4.6
17	None	0	N	95	95	20, 80%	B	5.0, 5.0
18	HA	2	17	90	95	10, 90%	B	5.2, 5.2
19	None	3	N	90	90	0, 100%	R	5.0, 5.0
20	None	0	N	104	207	0, 100%	R	4.0, 4.1
21	HA	0	19	100	None	5, 95%	B	5.0, 5.0
22	HA	3	14	100	99	40, 60%	R	5.0, 5.0
23	HA	0	5	80	100	10, 90%	L	5.1, 5.0
24	None	0	N	120	120	0, 100%	L	5.1, 5.0
25	HA	1	5	90	95	0, 100%	R	4.6, 4.7
26	HA	0	7	70	75	75, 25%	R	4.8, 4.9
27	HA	3	3	95	100	70, 30%	L	5.0, 5.0
28	HA	1	1	95	105	90, 10%	L	4.4, 4.1

Stimulation: HA, hearing-aid; CI, cochlear implant; None, Not wearing a hearing aid; L, left; R, right; B, bilateral.

### 2.2 Stimuli

**Figure 1:**
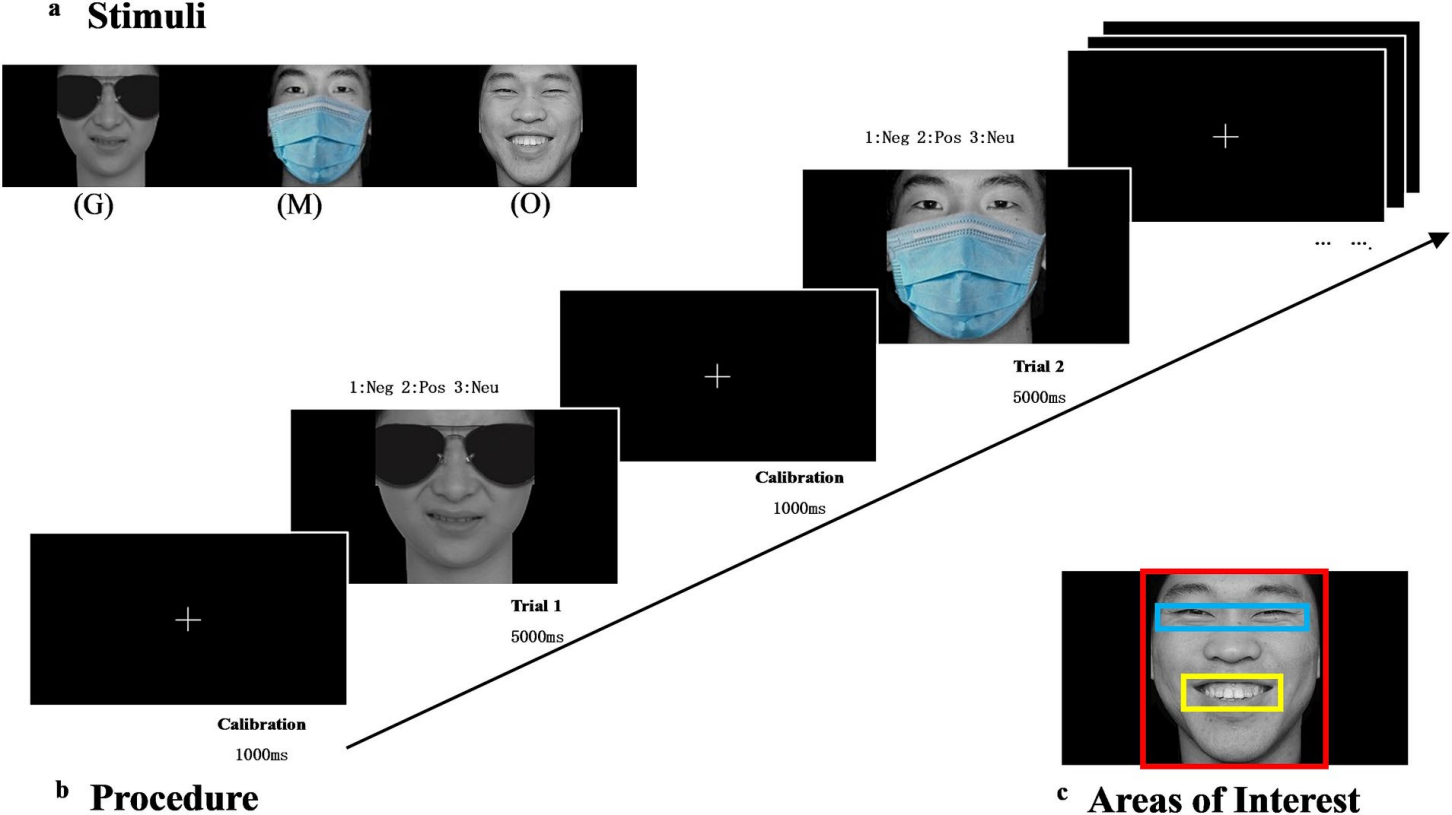
Stimuli and experimental procedure. **(a)** Stimuli: G, sunglasses; M, mask; O, original face. **(b)** Experimental procedure: Neg, negative; Neu, neutral; Pos, positive. **(c)** Areas of Interest (AOI): Three sets of AOIs were created to analyze the ocular exploration. Blue area: eye, Yellow area: mouth, Red area: the whole face. Adapted with permission from Gong et al. (2011).

This study sought to improve the authenticity of the stimuli by employing the approach introduced by Kim et al. (2022) instead of covering the face with bubbles or segmenting specific facial regions. The experimental materials were selected from the Chinese Affective Picture System (CAPS; Gong et al., 2011), comprising 90 images across three emotional categories (positive, neutral, and negative). Given the limitations of the dataset, a sufficient number of images depicting a single negative emotion to match the other categories were unavailable. To maximize ecological validity within these constraints, multiple discrete negative emotions were included (sadness: *n* = 9, anger: *n* = 8, fear: *n* = 7, disgust: *n* = 3, surprise: *n* = 3), with controls for several confounding factors, including arousal and gender. All selected images had arousal ratings above 4 on a 9-point scale, ensuring comparable emotional salience across categories. Statistical analysis confirmed no significant differences in arousal levels between emotional categories (one-way ANOVA: F[2,87] = 0.73, *p* = 0.48). The mean arousal ratings were 5.05 (SD = 0.19) for positive expressions (happiness), 5.03 (SD = 0.32) for negative expressions, and 4.96 (SD = 0.44) for neutral expressions (calmness). To address potential gender effects, balanced gender representation was maintained (15 male and 15 female faces) within each primary emotion category. Using Adobe Photoshop 2018, the original 90 facial images were modified by superimposing standardized sunglasses and masks, resulting in 90 images featuring sunglasses and another 90 images featuring masks, as illustrated in Figure 1a. This process generated a total of 270 facial images, comprising 3 (emotion: positive, neutral, negative) × 3 (occlusion: sunglasses, mask, original face) × 30 (individuals). This methodological approach ensures rigorous control over variables that could influence participants’ emotion recognition accuracy and reaction times, thereby enhancing the internal validity of our study.

### 2.3 Procedure

The experiment was conducted in a quiet laboratory using a Hasee K610D-i7 D4 laptop with a 15.6-inch screen, a resolution of 1920 × 1,080, and a refresh rate of 60 Hz. A Tobii Nano Pro eye tracker with a sampling rate of 60 Hz and a chin rest to stabilize participants’ heads were also used. E-Prime 3.0 software was used for eye-tracking calibration, stimulus presentation, and data recording. Participants were engaged in an emotion judgment task, which required them to rapidly identify the emotional attribute of the stimulus image and provide a response by pressing a designated key within a specified time frame. Before the experiment, participants underwent a 9-point calibration to ensure precise recording of their eye movements. During the experiment, participants were seated 60 cm from the screen.

The experimental procedure is depicted in Figure 1b. First, a white fixation cross (+) was displayed centrally on a black computer screen for 1,000 milliseconds. Next, the stimulus image was shown in the center of the screen for 5,000 milliseconds, and participants were required to press a key to complete the emotion judgment task (negative: 1, positive: 2, neutral: 3). Following the participant’s keypress response, the facial stimulus immediately disappeared from the screen. If no response was made within the 5,000-ms time window, the face stimulus was automatically removed, and the trial terminated. In both scenarios, the experiment subsequently advanced to the next trial, reverting to a black background with a white cross displayed at the center. A total of 270 trials were presented in a random order without controlling for the sequence of occluded versus unoccluded stimuli. The entire experiment took approximately 40 min. Prior to the formal experiment, participants completed 30 training trials to acclimate the procedure.

### 2.4 Data analysis

The experiment collected both behavioral and eye-tracking data from the participants. Behavioral data included reaction times for correct responses and accuracy rates for each participant. RT outliers were identified and removed using a ± 2 SD cutoff from each participant’s mean reaction time. Eye-tracking data consisted of the percentage of time spent gazing at the eye region (blue area) and the mouth region (yellow area) relative to the total face region (red area), as illustrated in Figure 1c. Statistical analyses were conducted using R, with visualizations generated through the ggplot2 package (Wickham, 2016). We conducted four separate three-way mixed-design analyses of variance (ANOVAs) to evaluate the effects of participant group, stimuli type, and their interactions. The dependent variables analyzed were reaction time, accuracy rate, and the proportion of time spent gazing at the mouth and eye regions. For all parameters, the assumption of homogeneity of variance was verified using Levene’s tests, and *p*-values were corrected for multiple comparisons via the Bonferroni correction.

## 3 Results

### 3.1 Behavioral results

Table 2 presents the mean and standard deviation of reaction times and accuracy rates for deaf and hearing participants under different occlusion types (sunglasses, mask, original face) and emotion types (positive, neutral, negative).

**Table 2:** Descriptive statistics for reaction times and accuracy ratings.

Group	Occlusion	Emotion	Time (ms)		Accuracy (%)	
			*M*	*SD*	*M*	*SD*
DP	G	Neg	1,377	398	52.25	18.28
		Neu	1,288	416	75.58	25.90
		Pos	1,106	326	84.40	19.60
	M	Neg	1,422	426	58.24	18.61
		Neu	1,400	477	76.35	25.35
		Pos	1,419	437	49.31	15.82
	O	Neg	1,341	449	73.07	17.99
		Neu	1,333	396	76.51	24.84
		Pos	1,158	346	83.63	16.51
HP	G	Neg	1,300	264	67.23	16.54
		Neu	1,241	290	84.82	14.36
		Pos	1,143	320	86.13	13.20
	M	Neg	1,352	262	66.04	14.45
		Neu	1,330	333	84.23	17.20
		Pos	1,409	290	45.16	19.59
	O	Neg	1,190	211	87.90	8.67
		Neu	1,259	278	83.42	15.78
		Pos	1,123	240	86.63	14.65

Total sample size: *N* = 62; DP, deaf people; HP, hearing people; G, sunglasses; M, mask; O, original face; Neg, negative; Neu, neutral; Pos, positive.

#### 3.1.1 Results of ANOVAs on reaction times

With participant group, occlusion, and emotion as independent variables, and reaction times as the dependent variable, a three-way mixed-design analysis of variance (ANOVA) was conducted. Results showed that there were significant main effects related to occlusion, *F(2,118) = 59.034, p < 0.001,η2 p = 0.500*, and emotion, *F(2,118) = 8.065, p < 0.001,η2 p = 0.120*. Moreover, the interaction effect between occlusion and emotion was significant, *F(4,236) = 18.254, p < 0.001,η2 p = 0.236*.

**Figure 2:**
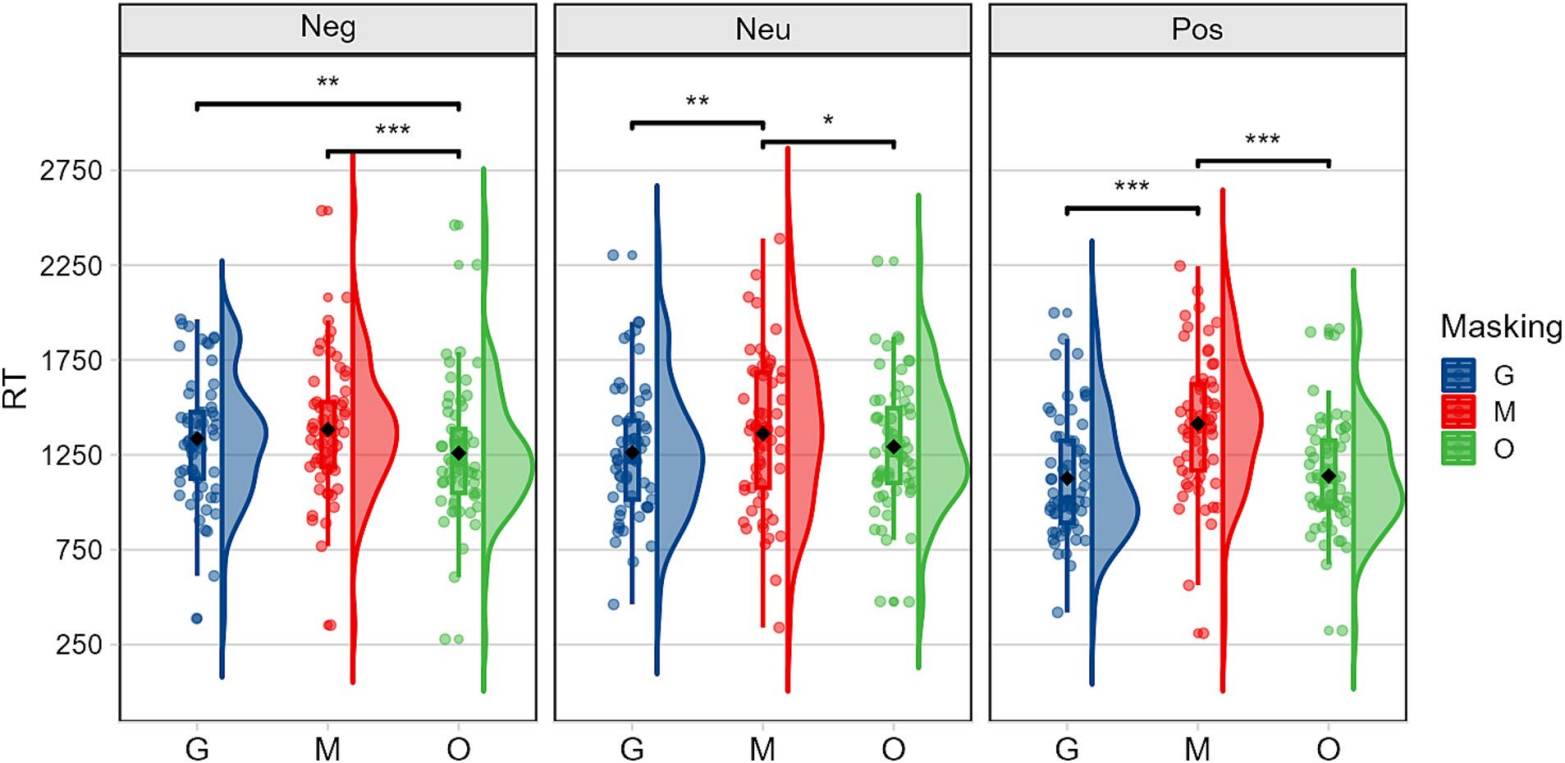
Reaction times illustrating the occlusion (G, sunglasses; M, mask; O, original face) × emotion (Neg, negative; Neu, neutral; Pos, positive) interaction.

Given the significant interaction effect between occlusion and emotion, simple effects analyses were conducted to examine the effect of occlusion at each level of emotion. Results indicated that the effect of occlusion was significant across all emotional conditions: for positive emotions, *F(2,59) = 61.139, p < 0.001,η2 p = 0.675*, for neutral emotions, *F(2,59) = 5.797, p = 0.005,η^2^ p = 0.164*, for negative emotions, *F(2,59) = 10.910, p < 0.001,η2 p = 0.270.* Bonferroni-corrected pairwise comparisons revealed that across all emotional valences (positive, neutral, negative), reaction times were significantly longer for masked faces compared to both sunglasses and original face conditions. As shown in Figure 2, this pattern was most pronounced for positive emotions (*MD_M-G_* = 289 ms, *MD_M-O_* = 274 ms). Moreover, t the effect of emotions was significant under both sunglasses (*F(2,59) = 17.090, p < 0.001,η2 p = 0.367*) and original face conditions (*F(2,59) = 14.316, p < 0.001,η2 p = 0.327*). Bonferroni-corrected pairwise comparisons further revealed that, under both sunglasses and original face conditions, reaction times for positive emotions were significantly shorter than those for negative and neutral emotions, suggesting that participants identified positive emotion faces more quickly.

#### 3.1.2 Results of ANOVAs on accuracy

The ANOVA results of accuracy revealed significant main effects for participant group *F(1,59) = 6.325, p = 0.015,η2 p = 0.097*, occlusion *F(2,118) = 195.678, p < 0.001,η^2^_p_ = 0.768,* and emotion *F(2,118) = 12.243, p < 0.001,η^2^_p_ = 0.172.* The interaction effects showed that all pairwise interactions between the factors were significant: the interaction between participant group and occlusion was significant *F(2,118) = 3.910, p = 0.023,η^2^_p_ = 0.062*; the interaction between group and emotion was marginally significant *F(2,118) = 2.923, p = 0.058,η^2^_p_ = 0.047;* and the interaction between occlusion and emotion was highly significant *F(4,236) = 86.601, p < 0.001,η^2^_p_ = 0.595.*

**Figure 3:**
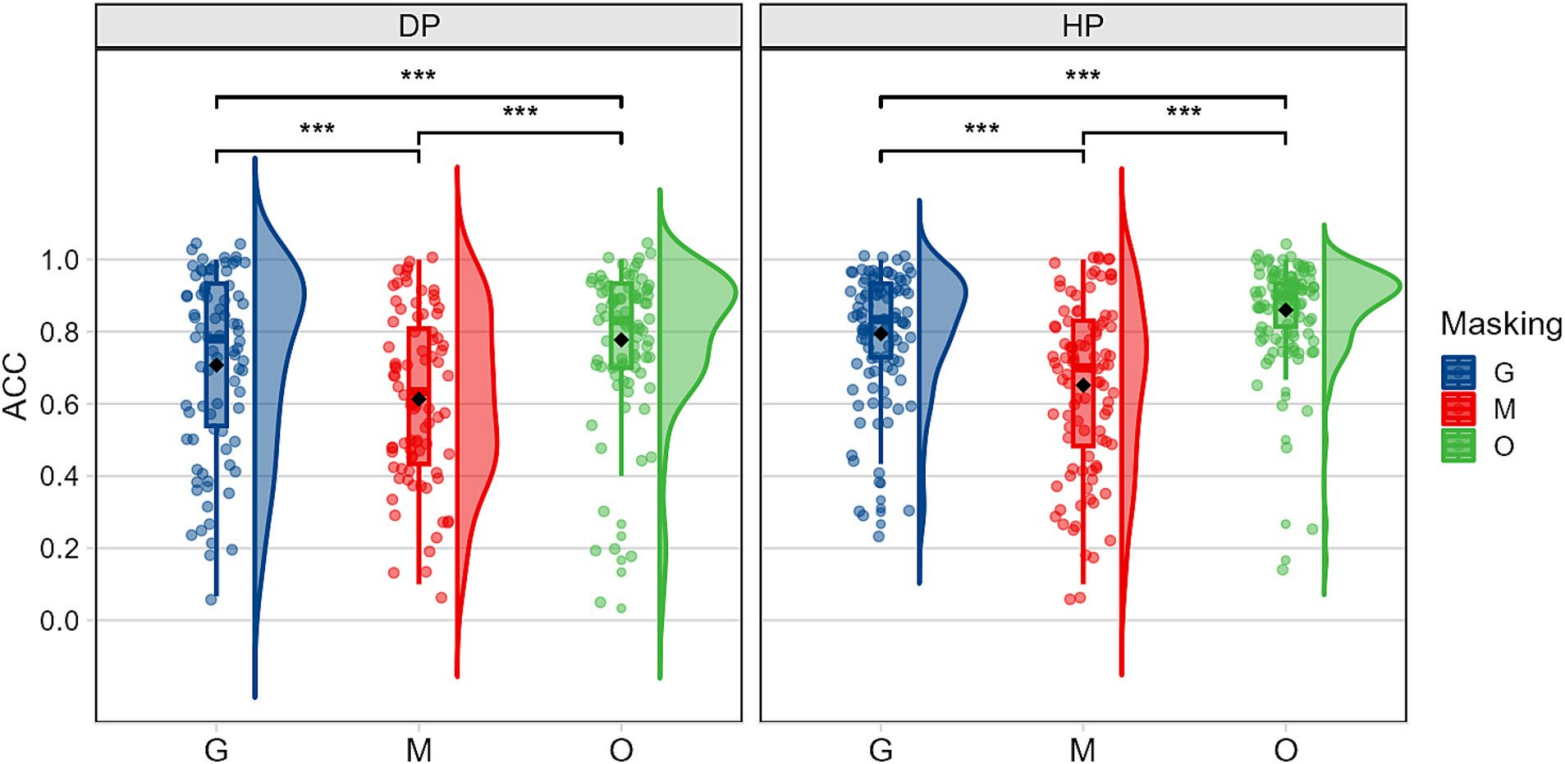
Accuracy rates illustrating the group (DP: deaf people, HP: hearing people) × occlusion (G, sunglasses; M, mask; O, original face) interaction.

Simple effects analyses were conducted to examine the interaction between participant group and occlusion. As shown in Figure 3, under sunglasses condition, the simple effect of group was significant, *F(1,59) = 8.664, p = 0.005,η2 p = 0.128*; under original face conditions, the simple effect was also significant *F(1,59) = 6.517, p = 0.013,η2 p = 0.099*, with deaf participants having lower accuracy rates compared to hearing participants. For both deaf and hearing participants, the simple effects across the three occlusion conditions were significant *F(2,59) = 58.112, p < 0.001,η2 p = 0.663*; *F(2,59) = 101.745, p < 0.001,η2 p = 0.775*. Bonferroni-corrected pairwise comparisons further revealed that recognition accuracy was lowest under mask conditions, followed by sunglasses conditions, with original face conditions yielding the highest recognition accuracy.

Simple effects analyses of the interaction between Group and Emotion revealed that, compared to hearing individuals, deaf individuals had a significantly lower recognition rate for negative emotions *F(1,60) = 14.657, p < 0.001,η2 p = 0.196*, while no significant differences were found between the two groups in the recognition of neutral and positive emotions. When analyzing the main effect of emotion type with group as a moderating variable, both deaf participants *F(2,60) = 7.960, p < 0.001,η2 p = 0.210* and hearing participants *F(2,60) = 5.762, p = 0.005,η2 p = 0.161* showed significant differences in accuracy across different emotion conditions. Pairwise comparisons further clarified these patterns: deaf participants demonstrated significantly lower accuracy in recognizing negative emotions compared to neutral and positive emotions. In contrast, hearing participants exhibited the lowest accuracy for positive emotions and the highest accuracy for neutral emotions.

**Figure 4:**
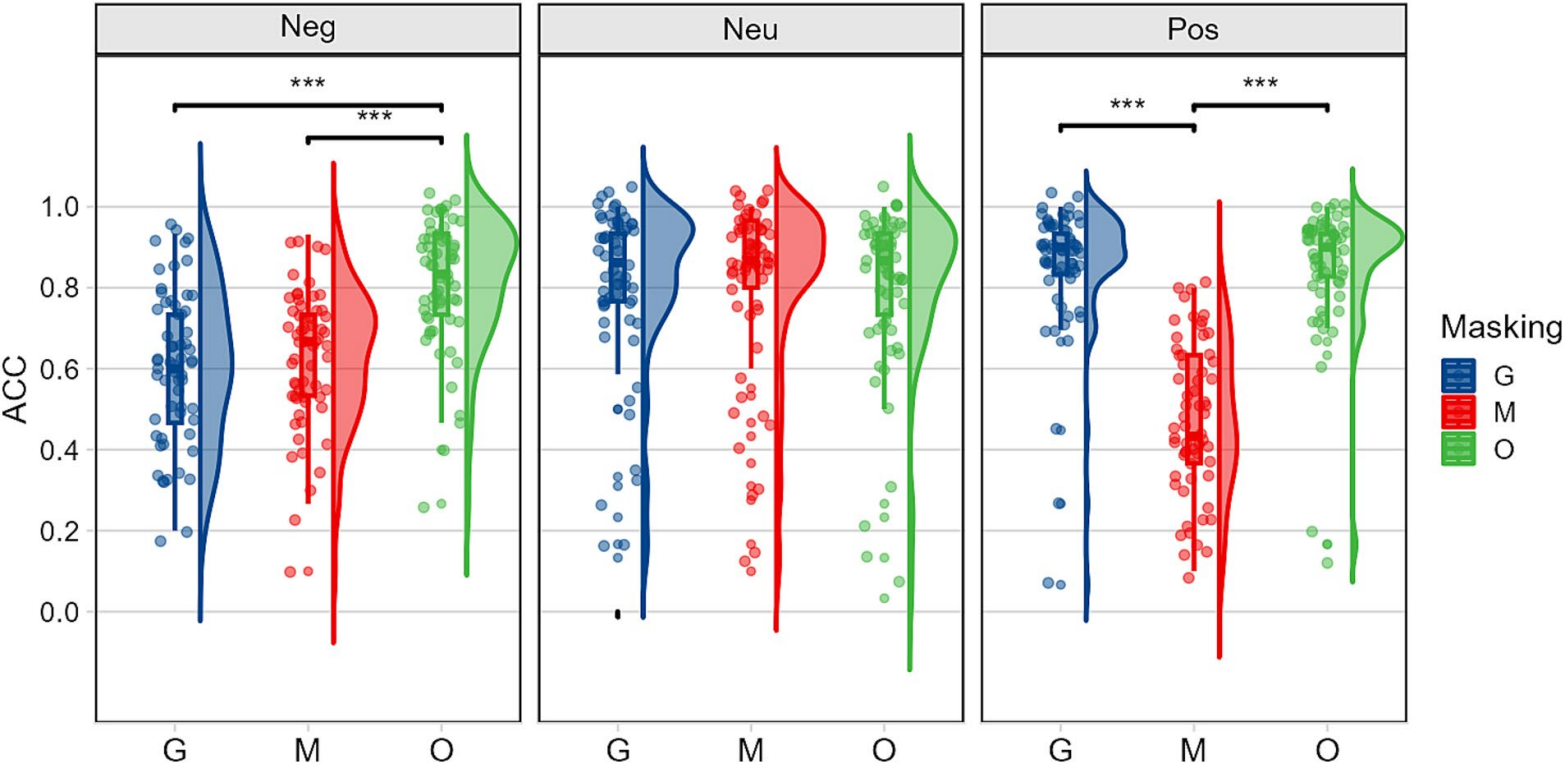
Accuracy rates illustrating the occlusion (G, sunglasses; M, mask; O, original face) × emotion (Neg: negative, Neu: neutral, Pos: positive) interaction.

As shown in Figure 4, simple effects analyses of the occlusion and emotion interaction showed significant effects for emotion under sunglasses *F(2,59) = 34.763, p < 0.001,η2 p = 0.541*, mask *F(2,59) = 35.986, p < 0.001,η2 p = 0.550*, and original face conditions *F(2,59) = 3.933, p = 0.025,η2 p = 0.118*. Pairwise comparisons revealed that under sunglasses, the recognition rate for negative emotions was significantly lower than for the other two types of emotions. Under mask conditions, the recognition rate for positive emotions was the lowest, followed by negative emotions, with neutral emotions being recognized the most accurately. Under original face conditions, neutral emotions had the lowest recognition rate, while positive emotions had the highest. From the perspective of emotion, the simple effect of occlusion was significant for recognizing negative emotions *F(2,59) = 158.177, p < 0.001,η2 p = 0.843*, and positive emotions *F(2,59) = 124.861, p < 0.001,η2 p = 0.809*, not significant for neutral emotions. Pairwise comparisons showed that, for negative emotions, the recognition accuracy was significantly higher for original faces compared to sunglasses and mask conditions, while for positive emotions, the accuracy was highest under sunglasses and lowest under mask. These results underscore the importance of the mouth in recognizing positive emotions and the eyes in recognizing negative emotions.

### 3.2 Results of eye-tracking

Table 3 presents the percentage of time spent fixating on the eye and mouth regions for both deaf and hearing participants under various occlusion and emotion conditions.

**Table 3:** Descriptive statistics for percentage of fixation time on the eye and mouth areas of interest.

Group	Occlusion	Emotion	Eye ratio (%)		Mouth ratio (%)	
			*M*	*SD*	*M*	*SD*
DP	G	Neg	3.55	6.12	25.50	13.39
		Neu	2.20	3.69	22.38	15.55
		Pos	2.13	3.25	25.94	14.70
	M	Neg	48.80	17.70	0.68	0.87
		Neu	53.56	18.28	0.75	0.87
		Pos	56.23	19.50	0.52	0.55
	O	Neg	26.99	19.15	7.20	6.63
		Neu	29.18	20.46	6.64	7.47
		Pos	22.30	19.01	9.20	7.23
HP	G	Neg	2.21	3.09	36.78	15.30
		Neu	2.13	2.95	34.39	16.75
		Pos	1.73	3.09	37.37	17.05
	M	Neg	51.04	12.63	0.49	0.52
		Neu	56.46	14.71	0.39	0.40
		Pos	60.56	12.08	0.34	0.40
	O	Neg	23.94	13.70	12.23	9.48
		Neu	27.13	15.73	11.52	10.23
		Pos	21.46	14.25	14.27	12.78

Total sample size: *N* = 62; DP, deaf people; HP, hearing people; G, sunglasses; M, mask; O, original face; Neg, negative; Neu, neutral; Pos, positive.

#### 3.2.1 Fixation results of the eye area

Among the three independent factors, significant main effects were observed for occlusion, *F(2,118) = 422.833, p < 0.001,η2 p = 0.878*, and emotion, *F(2,118) = 14.748, p < 0.001,η2 p = 0.200*. Specifically, the amount of time spent fixating on the eye region was ranked from longest to shortest as follows: mask condition > original face > sunglasses condition; neutral emotion > negative emotion > positive emotion. Regarding interaction effects, the interaction between occlusion and emotion was highly significant, *F(4,236) = 68.511, p < 0.001,η2 p = 0.537*, while other interactions were not significant.

**Figure 5:**
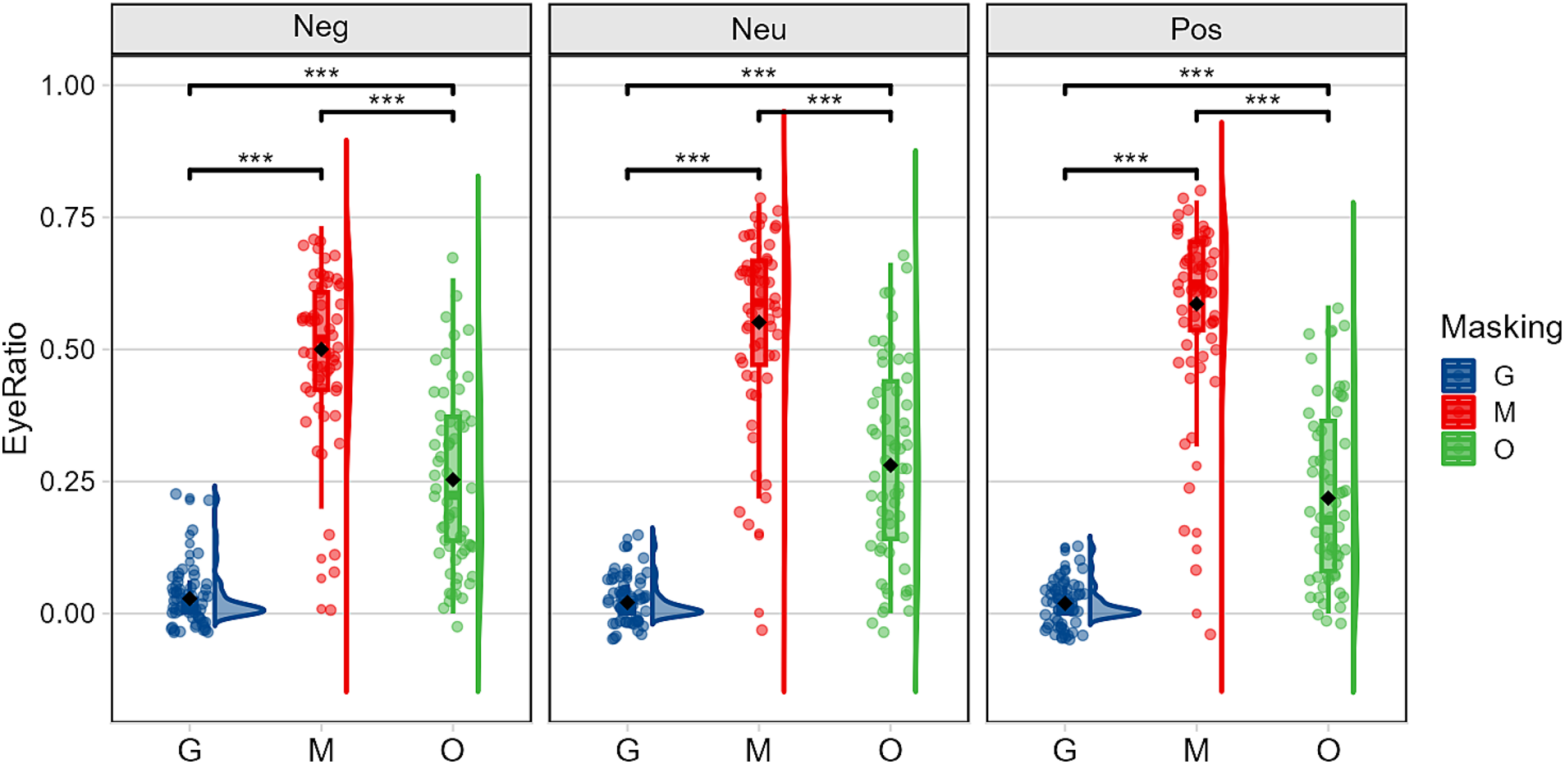
Mean (± standard error) of the proportion of time spent in the eye area illustrating the occlusion (G, sunglasses; M, mask; O, original face) × emotion (Neg, negative; Neu, neutral; Pos, positive) interaction.

Simple effects analyses of the interaction between occlusion and emotion revealed significant effects for emotion at different occlusion types, as illustrated in Figure 5. Under the mask condition, the simple effect of emotion was significant, *F(2,59) = 72.543, p < 0.001,η2 p = 0.711*, the rank for visual attention participants devoted to the eye region was positive emotions > neutral emotions > negative emotions. Under the original face condition, the simple effect of emotion was also significant, *F(2,59) = 22.290, p < 0.001,η2 p = 0.430*. Pairwise comparisons revealed that attention to the eye region was neutral emotions > negative emotions > positive emotions. Besides, despite the eyes being obscured under sunglasses conditions, deaf individuals still allocated some attention to the eye region, with the longest fixation times occurring for negative emotions. For the occlusion on different emotion conditions, the simple effects of fixation percentage on the eye region were also significant: under negative emotions, the simple effect of occlusion was significant, *F(2,59) = 356.914, p < 0.001,η2 p=. 924*; under neutral emotions, *F(2,59) = 358.660, p < 0.001,η2 p=. 924*; and under positive emotions, *F(2,59) = 431.512, p < 0.001,η2 p=. 936.* Pairwise comparisons further demonstrated that in all three emotion conditions, participants’ visual attention to the eye region was highest under the mask condition, followed by the original face condition, and lowest under the sunglasses condition.

#### 3.2.2 Fixation results of the mouth area

The results showed a significant main effect of group, *F(1,59) = 7.788, p = 0.007,η2 p = 0.117*, with hearing participants spending significantly more time fixating on the mouth region compared to deaf participants. The main effect of occlusion was also significant, *F(2,118) = 203.839, p < 0.001,η2 p = 0.776*, with the longest fixation times observed under the sunglasses condition, followed by the original face condition. Interestingly, the participants had also allocated some visual attention to the mouth region even under the mask condition. The main effect of emotion type was significant, *F(2,118) = 13.161, p < 0.001,η2 p = 0.182*. Post-hoc comparisons revealed that, for all participants, fixation times on the mouth region varied from longest to shortest as follows: positive emotions, negative emotions, and neutral emotions. Regarding interaction effects, the interaction between group and occlusion was significant, *F(2,118) = 7.690, p < 0.001,η2 p = 0.115*, as was the interaction between occlusion and emotion, *F(4,236) = 7.679, p < 0.001,η2 p = 0.115*.

**Figure 6:**
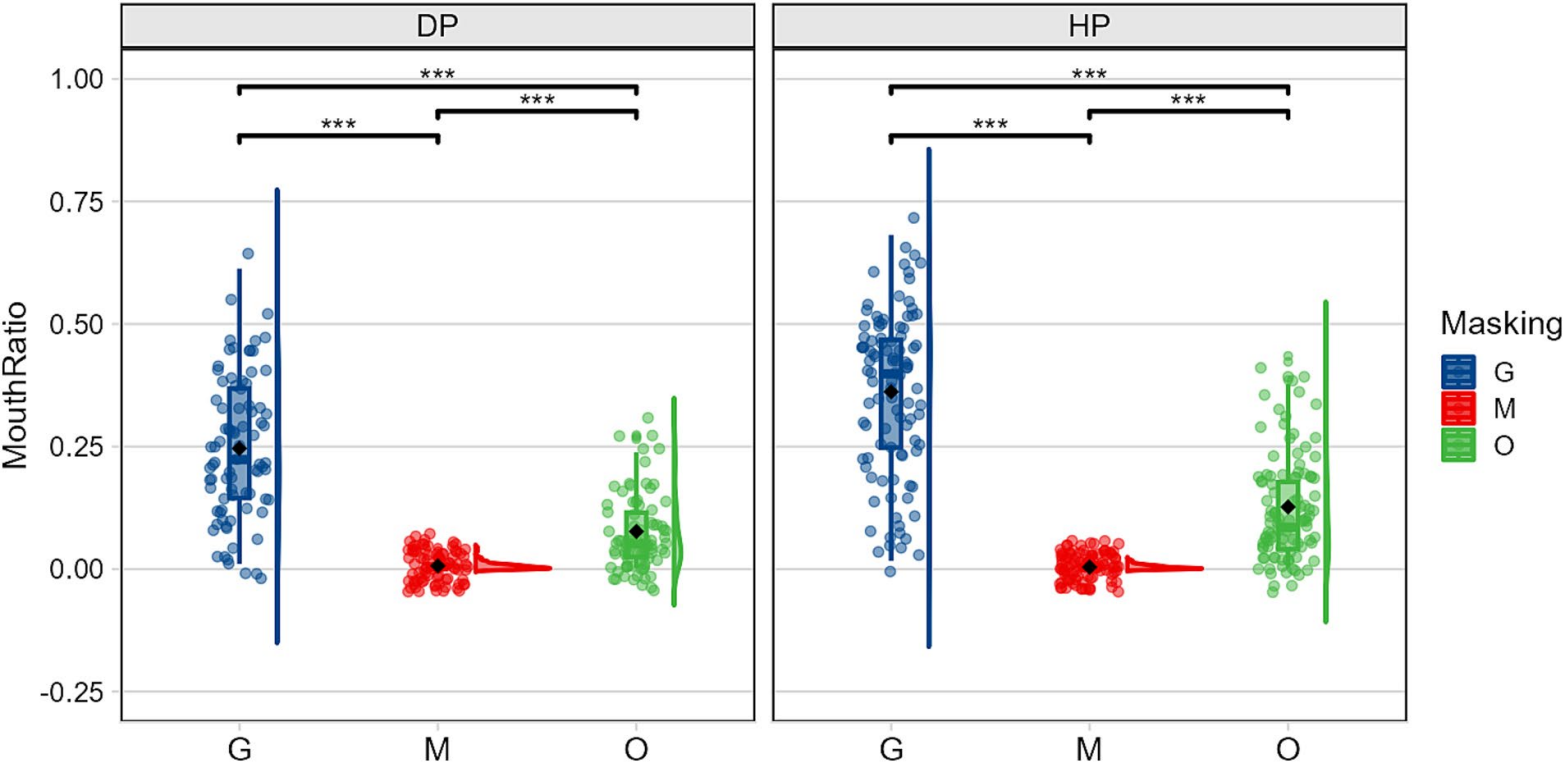
Mean (± standard error) of the proportion of time spent in the mouth area illustrating the group (DP, deaf people; HP, hearing people) × occlusion (G, sunglasses; M, mask; O, original face) interaction.

As illustrated in Figure 6, simple effects analyses of the interaction between group and occlusion revealed significant differences of group in the percentage of time spent fixating on the mouth region under both the sunglasses and original face conditions, *F(1,59) = 8.824, p = 0.004,η2 p = 0.130; F(1,59) = 4.678, p = 0.035,η2 p = 0.073*, with deaf participants spending significantly less time fixating on the mouth region compared to hearing participants. However, under the mask condition, deaf participants spent significantly more time fixating on the mouth region compared to hearing participants *F(1,59) = 4.922, p = 0.030,η2 p = 0.077*. Both deaf participants, *F(2,59) = 41.779, p < 0.001,η2 p = 0.586*, and hearing participants, *F(2,59) = 100.329, p < 0.001,η2 p = 0.773*, showed significant differences in fixation times on the mouth region for the three types of stimuli. The fixation times ranked from longest to shortest were: sunglasses condition > original face > mask condition.

**Figure 7:**
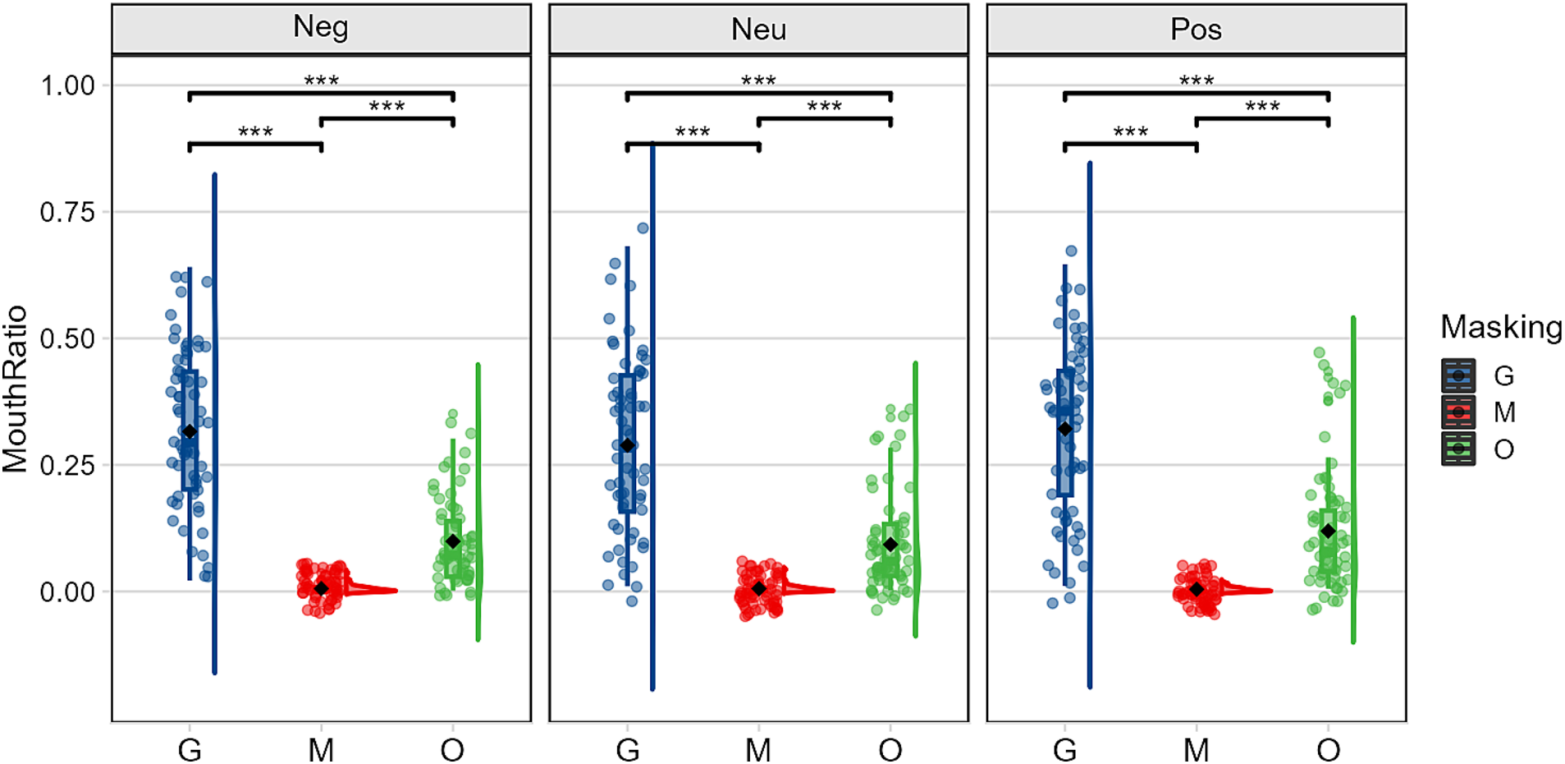
Mean (± standard error) of the proportion of time spent in the mouth area illustrating the occlusion (G, sunglasses; M, mask; O, original face) × emotion (Neg, negative; Neu, neutral; Pos, positive) interaction.

There were significant differences in the amount of time participants spent fixating on the mouth region for various emotions under different occlusion conditions (see Figure 7). Specifically, the simple effects of group were significant for both sunglasses, *F(2,59) = 11.305, p < 0.001,η2 p = 0.277*, and original face conditions, *F(2,59) = 8.492, p < 0.001,η2 p = 0.224*. Post-hoc comparisons showed that, regardless of the occlusion conditions, participants allocated the most visual attention to the mouth region for positive emotions, followed by negative emotions, and least for neutral emotions. This indicates that the mouth is a crucial area for recognizing positive emotions. Furthermore, the simple effects of occlusion type on the percentage of time spent fixating on the mouth region were also significant across different emotion conditions: negative emotions, *F(2,59) = 151.549, p < 0.001,η2 p=. 837*; neutral emotions, *F(2,59) = 101.421, p < 0.001,η2 p=. 775*; and positive emotions, *F(2,59) = 120.698, p < 0.001,η2 p =* 0*.804*. For all three emotion conditions, participants allocated more visual attention to the mouth region in the sunglasses condition compared to the original face and mask conditions.

## 4 Discussion

This study has explored the mechanisms underlying emotion recognition in deaf individuals under different facial occlusion conditions. Behavioral and eye-tracking results reveal the following four key findings: First, both sunglasses and masks significantly impair emotion perception in deaf individuals, though they can enhance it under certain conditions. Second, deaf individuals allocate more attention to the eye region when processing negative emotions compared to positive ones, yet their ability to recognize negative emotions is poorer. Third, masks have a greater impact on emotion recognition than sunglasses, with the mouth region being particularly crucial for identifying positive emotions. Finally, these results suggest that deaf individuals may experience both visual deficits and compensatory enhancements under different conditions, supporting the integration theory of visual function in the deaf population. Overall, these findings highlight the significant challenges faced by deaf individuals in recognizing negative emotions and processing emotion recognition under facial occlusion conditions.

The first finding underscores the significant impact of facial occlusions, particularly masks, on emotion recognition among deaf individuals. Compared to original face conditions, deaf individuals had lower accuracy and slower reaction times in emotion recognition under occlusion conditions. This aligns with previous research emphasizing the unique challenges faced by the deaf community in interpreting emotional cues, especially when visual access to facial features is restricted (Amadeo et al., 2022; Gutierrez-Sigut et al., 2022). However, occluding specific facial regions does not necessarily impair emotion recognition; rather, it may enhance the emotion recognition in certain circumstances. Our results showed that, under mask conditions, deaf individuals spent less time fixating on the eyes when recognizing negative emotions compared to positive ones. This suggests that the occlusion of the mouth region may facilitate the recognition of negative emotions. Covering the mouth may reduce distractions, enabling deaf individuals to more effectively capture and process subtle changes in eye expressions. Correspondingly, behavioral results also showed that under sunglasses conditions, participants recognized positive emotions with the highest accuracy and the fastest reaction times. This indicates that the occlusion of the eyes might reduce visual cognitive load, making the interpretation of mouth-related positive emotions quicker and more accurate for deaf individuals. These findings align with the notion that covering facial regions less relevant to emotion recognition may help filter out irrelevant or misleading information (Leach et al., 2016). It also suggests that facial occlusion may serve as an effective strategy to help deaf individuals more accurately understand others’ emotional states in complex social interactions.

The second finding indicates that the eye region is the most critical area for recognizing negative emotions, and deaf individuals exhibit superiority effect of happiness. Under both sunglasses and original face conditions, deaf individuals allocated more attentional resources on negative emotions than positive ones in the eye region, suggesting deaf individuals have an advantage over recognizing positive emotions from the eyes as compared with negative emotions. This result replicates the superiority effect of happiness (Amadeo et al., 2022), that happy faces were typically recognized most quickly in emotion classification tasks (Nummenmaa and Calvo, 2015). The advantage, better performance on recognizing positive emotions, may be related to the current preferable social environment. Social improvements in areas such as family-centered care (Hlayisi and Sekoto, 2023), peer support (Wang et al., 2023), and technological support (Chapman et al., 2023) have afforded deaf individuals greater exposure to positive emotional expressions in social interactions. On the other hand, this interpretation also aligns with the latest evidence suggesting that deaf individuals may have insufficient social experience with expressing and recognizing negative emotions, which can lead to increased difficulty in recognizing them (Tsou et al., 2021). Additionally, the lower sensitivity to negative emotions among deaf individuals may also be related to their tendency to avoid conflicts and disputes in social environments (Yuen et al., 2022).

The third finding highlights the crucial role of mouth in conveying positive emotions (Beaudry et al., 2014; Bombari et al., 2013; Schurgin et al., 2014). However, the occlusion caused by mask severely restricts deaf individuals’ ability to derive emotional information from the mouth area, thereby compelling them to rely more heavily on other facial features for emotion recognition, particularly the eyes. In particular, participants exhibited better performance under mask conditions than under sunglasses conditions. Moreover, the proportion of fixations on the mouth region under mask conditions was significantly lower than that of fixations on the eye region under sunglasses conditions. This suggests that the occlusion of the mouth seems to have a greater impact on facial emotion recognition compared to the eye occlusion. While core linguistic comprehension remained intact, the ability to attribute emotions and attitudes was compromised when the lower face was obscured (Giovanelli et al., 2023). This may be attributed to the fact that mask covers a larger portion of the face compared to sunglasses (Carbon, 2020). In occlusion conditions, hearing individuals can rely on background sounds, voice tones, and intonation to compensate for the lack of facial expressions (Leitzke and Pollak, 2016). However, deaf individuals in the current study exhibited underdeveloped spoken language abilities due to hearing impairment or technical shortcomings of hearing device. They might experience difficulties to utilize auditory cues and rely more on visual cues to compensate for communication deficits. Therefore, it is crucial to find effective solutions to address this issue, such as developing transparent masks or enhancing other visual cues.

Taken together, the current findings demonstrate that deaf individuals may encounter visual deficits and compensatory enhancement under different conditions, aligning with the integration theory of visual function in deaf individuals (Dye and Bavelier, 2010). On the one hand, the behavioral and eye movement data indicate that deaf individuals have poorer overall facial emotion recognition abilities than hearing individuals, aligning with previous research indicating that deaf individuals may experience delays in facial emotion recognition (Wang et al., 2011). While deaf individuals had slightly longer total reaction times compared to hearing individuals, a notable difference was that they also spent less time fixating on both the eyes and mouth areas. This suggests that hearing individuals initially invest more attention resources in these key facial features, whereas deaf individuals tend to distribute their attention more widely, including peripheral areas. This may be due to the absence of auditory input, which leads deaf individuals to alter the scope of allocating visual attention, resulting in a more widespread distribution of their visual attentional resources (Smith et al., 1998). Furthermore, deaf individuals are less inclined to adjust their visual attention strategy based on task demands, whereas hearing individuals can flexibly allocate their visual resources according to experimental requirements.

On the other hand, we found that deaf individuals exhibited a compensatory enhancement in their visual perception for emotion recognition. Despite overall lower accuracy rates, deaf individuals had faster reaction times for positive emotions. Although deaf individuals showed more off-target fixations than hearing individuals, their recognition of positive emotions were not affected. This discrepancy might be due to the broader peripheral visual span and higher motion detection ability in deaf individuals (Shiell et al., 2014; Stevens and Neville, 2006), enabling them to detect information from the mouth area through peripheral vision while focusing on the eyes. Early hearing loss results in the lack of auditory stimuli during cortical development, which could lead to a reorganization of other modalities, such as vision. Then, the additional involvement, often referred to as “cross-modal reorganization,” of auditory cortices activated by visual stimuli might enhance deaf individuals’ ability to process visual information, potentially conferring a visual advantage in the recognition of certain stimuli (Benetti et al., 2021; Finney et al., 2001; Zhang et al., 2021). In other words, deaf individuals might develop stronger attention and faster visual processing speeds through heightened sensitivity to visual cues, allowing them to quickly identify and react to certain emotional signals as a compensation for their hearing loss.

Besides, this study has several limitations that future research should address. First, the stimuli used in this experiment were static images, which differed from real-life social interactions. Given that some studies suggest that deaf individuals may have advantages in dynamic emotion recognition, future research could employ dynamic audiovisual stimuli encompassing a broader range of emotional types to enhance ecological validity and generalizability. Second, our study categorized emotions into three types: positive, neutral, and negative, without further differentiation. Future research should include a wider variety of facial emotion types to thoroughly investigate the impact of facial occlusion on emotion recognition in deaf individuals. Third, the group of deaf participants in the current study is not homogeneous, as differences exist in language modality (sign language vs. oral language). Sign language, as a visual language, conveys speech information through the location and movement of gestures. The frequency of sign language use can lead to varying patterns of visual attention distribution among deaf individuals (Giovanelli et al., 2023; Wang et al., 2020). Therefore, future studies should carefully control for this factor to more accurately verify the effects of these variables. Finally, facial emotion recognition is not the sole channel for emotion identification; vocal cues, body language, and social context are also commonly used to analyze and interpret emotions. Future research could develop more effective assistive tools and training methods to enhance their emotion recognition abilities across different contexts. An effective way is to use transparent masks. However, Atcherson et al. (2021) found that transparent options have greater attenuation, resonant peaks, and deflect sounds in ways that non-transparent masks do not. Therefore, innovative solutions and technologies must be explored further to enhance social interactions within the deaf community, thereby improving their quality of life and fostering greater social participation.

## 5 Conclusion

This study utilized eye-tracking technology to investigate differences in facial emotion perception between deaf and hearing individuals under various facial occlusion conditions. From both behavioral and eye-tracking data, the current study reveals that, deaf individuals exhibited weaker emotion recognition abilities compared to hearing individuals across most facial occlusion conditions, but they performed better in recognizing positive emotions. This suggests that deaf individuals experience both deficit and compensation of visual function, but these phenomena occur under different conditions, supporting the integration theory proposed by Dye and Bavelier. Additionally, facial occlusion (e.g., mask and sunglasses) significantly impacts the performance of emotion recognition in deaf individuals. Future studies should place more emphasis on the role of facial visual cues in emotion perception for deaf individuals and implement effective measures to improve this situation, such as promoting the use of transparent masks, enhancing emotional education for deaf children, and developing assistive technologies based on visual and tactile cues. These efforts could support the mental health and social integration of deaf individuals, contributing to the creation of a more inclusive and accessible social environment.

## Data Availability

The raw data supporting the conclusions of this article will be made available by the authors, without undue reservation.

## Footnotes

^1^Thank the reviewer for the concern regarding the heterogeneity of the deaf group. Although all deaf participants are prelingually deaf people and L1 speakers of Sign language, the usage ratios of spoken Chinese to Chinese Sign language could influence face processing abilities, which should be controlled in future research.
